# Percutaneous microwave ablation of bone lesions: a retrospective cohort study

**DOI:** 10.1186/s13244-025-02083-6

**Published:** 2025-09-17

**Authors:** Sylvain Bodard, Amgad M. Moussa, Max Vaynrub, Meredith Bartelstein, Ernesto Santos-Martin, Majid Maybody, Francois H. Cornelis

**Affiliations:** 1 Department of Radiology, Memorial Sloan Kettering Cancer Center, New York, NY USA; 2 Department of Surgery, Memorial Sloan Kettering Cancer Center, New York, NY USA; 3 Weill Cornell Medical College, Medicine, New York, NY USA

**Keywords:** Radiology (interventional), Microwaves, Bone neoplasms, Cancer pain

## Abstract

**Purpose:**

To evaluate the feasibility, safety, and efficacy of microwave ablation (MWA) of bone lesions with regard to local control and pain palliation.

**Materials and methods:**

We reviewed 43 patients (23 males, 20 females) with 51 lesions (44 metastatic, 7 benign) treated with MWA from January 2016 to December 2023. Pain intensity was measured using the Visual Analogue Scale (VAS), SF-36 Bodily Pain Scale, and Patient Global Impression of Change (PGIC) from pre-operation to various follow-up stages. Adverse events were categorized according to the Society of Interventional Radiology (SIR) grading system.

**Results:**

The procedure demonstrated 100% technical success. Grades I and III adverse events were observed in 8.3% (3/36) and 2.8% (1/36) of patients with metastatic disease, respectively. In those with benign lesions, no adverse events were reported. A significant reduction in pain was observed, with the VAS score decreasing by 74.3% from baseline to the last follow-up [6.7 ± 2.3 (range: 0–10) to 1.8 ± 2.3 (range: 0–7) (*p* < 0.001)] for metastatic patients, and from 5.7 ± 2.1 (range: 3–8) to 0 ± 0 (range: 0–0) by the final follow-up (*p* = 0.0011) for benign lesions. 77.8% (29/36) of metastatic patients, and all (7/7) benign patients were much or very much improved according to Patient Global Impression Change. Complete imaging response was achieved in 55.6% (20/36) of metastatic lesions. At last follow-up, 25% (9/36) had radiological evidence of recurrence, with a median recurrence time of 13 months (IQR: 8–14). Complete response was achieved in all benign lesions.

**Conclusions:**

MWA is a safe and effective treatment for pain management in patients with bone lesions.

**Critical relevance statement:**

This study confirms the potential of microwave ablation as a treatment for bone lesions, providing significant pain relief with a favorable safety profile.

**Key Points:**

- Microwave ablation (MWA) significantly reduced pain scores in patients with bone lesions, maintaining pain relief over time.
- The procedure exhibited high technical success with minimal adverse events, indicating a high safety profile.
- Subgroup analysis revealed no significant differences in pain reduction among different procedural combinations over time.

**Graphical Abstract:**

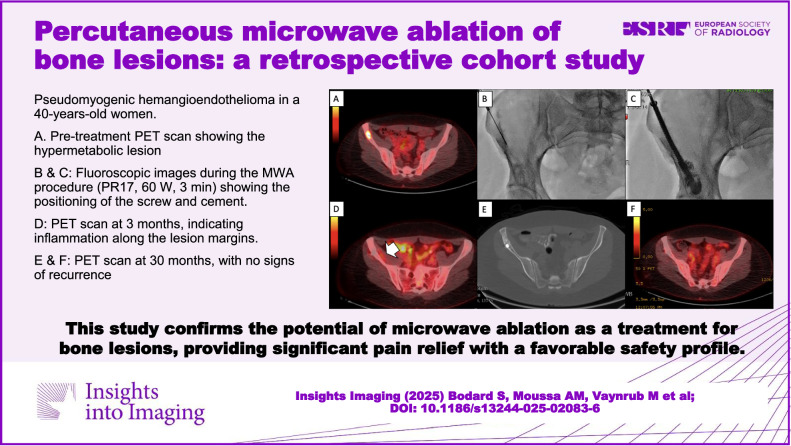

## Introduction

The objectives for patients with bone lesions are to concomitantly provide local control, alleviate symptoms, and preserve functional capabilities [1–3]. However, image-guided therapies in bone lack standardization in terms of technical approaches, detailed safety profiles, and evidence supporting the clinical outcomes of these techniques remain limited.

Compared to cryoablation (CA) or radiofrequency ablation (RFA), microwave ablation (MWA) has been less reported for local control and pain palliation [4, 5]. Some studies suggest that MWA may provide advantages in bone, such as shorter ablation time and less sensitivity to changes in bone impedance [6–9]. However, the theoretical disadvantages of MWA include less distinct ablation zone boundaries, potential nontarget overheating due to rapid delivery of high-power output, and poor reproducibility [10]. To provide more insights into the clinical use of MWA in bone, this retrospective study evaluates the feasibility, safety, and efficacy in terms of local control and pain palliation of MWA in the treatment of both benign and malignant bone lesions.

## Materials and methods

Ethical approval was granted by the hospital’s Ethics Committee for this retrospective, non-comparative cohort study (IRB #23-300). Informed consent was waived. This manuscript was prepared in accordance with the STROBE guidelines for reporting observational studies.

### Inclusion criteria

Inclusion criteria were as follows: (1) any bone tumor confirmed by pathology; and (2) percutaneous microwave ablation (NEUWAVE Microwave Ablation System) of one or more bone lesions, following the device’s Instructions for Use.

### Procedures

Four experienced interventional radiologists with 5 to 25 years of experience (A.M., F.C., E.S., M.M.) performed the procedures under imaging guidance (fluoroscopy, cone beam computed tomography, or CT scan). All procedures were performed under general anesthesia in the interventional (*n* = 36) or operative rooms (*n* = 7). The optimal trajectory was determined on preoperative CT scans. The configuration and size of the bone lesions, as well as their proximity to surrounding organs, were carefully evaluated in a multidisciplinary meeting. The needle was inserted along the pre-established path until the bone ablation needle accurately reached the target. Ablation parameters (adjusted by type of tumor and/or proximity to surrounding structures), such as power and duration, followed the recommendations to users provided by the company and were set to ensure that the ablation zone extended at least 5 mm beyond the lesion margin to achieve complete ablation. Patients at risk of bone fractures underwent osteoplasty, with or without additional fixation. After the procedures, the patients were monitored in the recovery room for 2 h.

### Follow-up and clinical evaluation

Postoperative pain related to bone lesions was assessed using the VAS and SF-36 Bodily Pain Scale at pre-operation, 1 day post-operation, 3 months post-operation, 6 months post-operation (except for benign lesions), and at the last follow-up. The VAS categorizes pain levels on a scale from 0 (no pain) to 10 (very severe pain), with intermediate scores defined as 1–3 for mild pain, 4–6 for moderate pain, and 7–9 for severe pain. Concurrently, the SF-36 Bodily Pain Scale classifies pain as None, Very Mild, Mild, Moderate, or Severe. These tools provided a comprehensive and standardized method for evaluating changes in pain intensity throughout the study. Additionally, the Patient Global Impression of Change (PGIC) scale was employed to gauge participants’ self-perceived changes in their condition. The PGIC scale includes the following categories: Very Much Improved, Much Improved, Minimally Improved, No Change, Minimally Worse, Much Worse, and Very Much Worse. This measure captures subjective improvements or deteriorations reported by patients. Adverse events (AEs) were graded using the guidelines of the Society of Interventional Radiology (SIR), which defines complications from Grade A to E based on their severity and clinical consequences. Grade A corresponds to events that require no therapy and have no consequence; Grade B includes events requiring nominal therapy without lasting effects; Grade C involves complications that require medical treatment or minor hospitalization (less than 48 h); Grade D includes those necessitating major therapy or prolonged hospitalization (more than 48 h); Grade E refers to adverse events resulting in permanent sequelae and Grade F refers to death [11]. In our study, adverse events were retrospectively classified based on clinical documentation and imaging, ensuring standardized and reproducible safety reporting. Tumor recurrence was monitored by enhanced CT and/or MRI. Local tumor recurrence was defined as an increase or peripheral expansion of osteolysis or the emergence of new enhanced nodules within or surrounding the ablation area on CT; on MR imaging, as a low signal on T1-weighted images and a high signal on T2-weighted images, with additional enhancement observed at the edge of or within the ablation cavity. Additionally, continuous uptake of fluorodeoxyglucose (FDG) on PET/CT scans was also considered a sign of recurrence. The last follow-up assessments were conducted in February 2024, which served as the data cut-off point for this study.

### Statistical analysis

Changes in pain scores were analyzed using paired samples *t*-tests, with the Shapiro–Wilk test assessing the normality of the score distributions. Differences between groups at various time points were tested using ANOVA, appropriate for comparing means across multiple groups when data distribution is assumed to be normal. Following the ANOVA, a Tukey post hoc test was performed to identify specific group pairs with significant differences. Statistical significance was determined at a *p*-value of less than 0.05. All analyses were performed using SPSS (Statistical Package for the Social Sciences) Version 26.0 (IBM Corp.).

## Results

### Patient and lesion characteristics

This retrospective study analyzed clinical data from 43 patients, 36 with metastases and 7 with benign bone tumors, who underwent non-vertebral MWA at our institution between January 2016 and December 2023. Of the 36 patients with metastases (17 males, 19 females), 29 (80.1%) had one target lesion, 4 (11.1%) had two, 2 (5.6%) had three, and 1 (2.8%) had four, totaling 44 metastases. Seven patients were treated intraoperatively during surgery performed by the orthopedic surgery team. Patients’ average age was 60 ± 13 years [range: 24–81], with a body mass index of 25 ± 7 [range: 14–53], and an Eastern Cooperative Oncology Group Performance Status Scale score of 1 ± 1 [range: 0–3]. Fourteen different primary cancers were identified, the most common being clear cell renal carcinoma (*n* = 10), thyroid cancer (*n* = 4), sarcomas (*n* = 4), and lung cancer (*n* = 3). Pain was the most common indication for treatment, with 94.4% of cases, followed by fractures or fracture risk (36.1%). Eight patients (22.2%) with solitary metastasis underwent treatment with a curative intent. Patients were indicated for ablation after multidisciplinary discussion when radiotherapy (for metastatic lesions) or surgery (for benign lesions) was not thought to be the preferred treatment modalities. The mean (± standard deviation (SD)) diameter of the metastases was 48 ± 27 mm [range: 15–130]. The most common sites were the pelvis (22 metastases), femur (12), and sternum (6). Mean (± SD) preoperative pain intensity on the VAS was 6.7 ± 2.3 [range: 0–10].

**Fig. 1:**
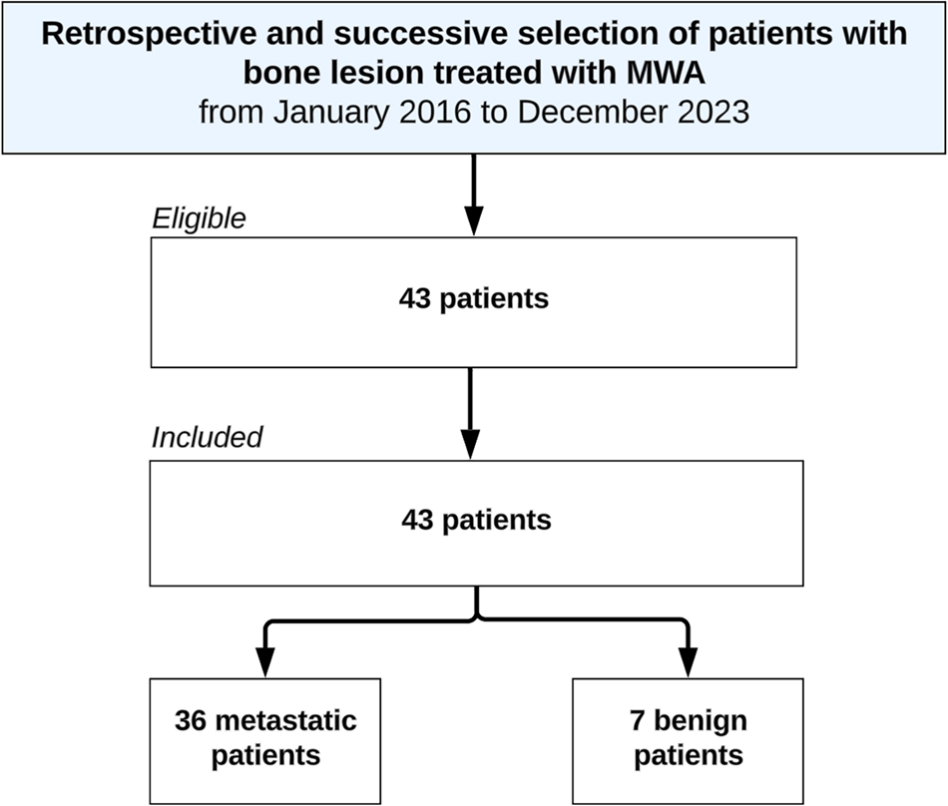
Study flowchart

Of the 7 benign patients included (6 males, 1 female), 6 (86%) had an osteoid osteoma, and 1 (14%) had an osteoblastoma. Patients’ mean (± SD) age was 19 ± 6 years [range: 11–30], with a body mass index of 25 ± 3 [range: 22–29]. The lesions were localized on the femur (*n* = 5), iliac bone (*n* = 1), and tibia (*n* = 1). The mean (± SD) tumor diameter was 11 ± 9 mm [range: 4–28]. The mean (± SD) preoperative pain intensity on the VAS was 6 ± 2 [range: 3–8]. Table 1 summarizes patient characteristics, Table 2 summarizes lesion characteristics, and Fig. 1 depicts the study flowchart.

**Table 1:** Patient characteristics

	Gender	17 M/19 F
Metastatic	Age at procedure (years) (mean, SD, range)	60 ± 13 (24–81)
	BMI (kg/cm^2^) (mean, SD, range)	25 ± 7 (14–53)
	ECOG PSS (mean, SD, range)	1 ± 1 (0–3)
	Primary cancer	
	Renal cell carcinoma	10
	Thyroid cancer	4
	Sarcoma	4
	Lung carcinoma	3
	Colon or rectal carcinoma	3
	Melanoma	3
	Pancreatic adenocarcinoma	2
	Hepatocarcinoma	1
	Breast cancer	1
	Prostate cancer	1
	Uterine leiomyosarcoma	1
	Dedifferentiated chondrosarcoma	1
	Pseudomyxogenic hemangioendothelioma	1
	Neuroendocrine tumor of unknown primary	1
	Number of target tumors	
	1	29
	2	4
	3	2
	4	1
	Patient indication of treatment	
	Pain	34/36
	Fracture or fracture risk	13/36
	Curative	8/36
Benign	Gender	6 M/1 F
	Age at procedure (years) (mean, SD, range)	19 ± 6 (11–30)
	BMI (kg/cm^2^) (mean, SD, range)	25 ± 3 (22–29)
	Lesion	
	Osteoid osteoma	6
	Osteoblastoma	1

*M* male, *F* female, *SD* standard deviation, *ECOG PSS* Eastern Cooperative Oncology Group Performance Status Scale, *VAS* Visual Analogue Scale

**Table 2:** Lesion characteristics

Metastatic	Size, maximum diameter (mm) (mean, SD, range)	48 ± 27 (15–130)
	Localization	
	Acetabulum/iliac bone	16
	Femur	12
	Sternum	6
	Ischial bone	3
	Pubic bone	2
	Humerus	3
	Ramus	1
	Tibia	1
Benign	Size, maximum diameter (mm) (mean, SD, range)	11 ± 9 (4–28)
	Localization	
	Femur	5
	Iliac bone	1
	Tibia	1

*SD* standard deviation, *mm* millimeter

### MWA procedures characteristics

Regarding the metastatic lesions, the average power delivered was 55 ± 25 watts [range: 30–140] with a mean total energy delivery time of 13 ± 11 min [range: 3–40] (Table 3). A total of 45 needles were used: 35 were 17 gauge (78%), 9 were 13 gauge (20%), and 1 was 15 gauge (2%). All lesions were treated with one needle, except for one lesion (two needles) due to its size and shape. Twenty patients (55.6%) underwent combined procedures. Osteoplasty was performed on 16 patients (44.4%) and, of these, 6 (16.7%) also received fixation. Additionally, 3 patients (8.3%) underwent embolization, and 1 patient (2.8%) received radiotherapy. Furthermore, biopsies were conducted during the procedure for 12 patients (33.3%). The average irradiation dose was 1431 ± 1719 mGy.cm [range: 196–6696] for CT-guided procedures and 52 ± 72 mGy.cm [range: 3–245] for fluoroscopy-guided procedures. The median hospital stay was 8 days [IQR: 1–31].

**Table 3:** MWA procedure characteristics

Metastatic	Type of needles	
	PR 15 or PR 20 (17 G)	33
	SR 25 (13 G)	7
	LK 15 (17 G)	2
	PR20XT (15 G)	1
	VT22 (13 G)	2
	Power (W) (mean, SD, range)	55 ± 25 (30–140)
	Total time energy delivery (min) (mean, SD, range)	13 ± 11 (3–40)
	Biopsy	12
	Other combined procedure (number of patients)	
	None	16
	Cementoplasty	10
	Cementoplasty + percutaneous screw fixation	6
	Embolization	3
	RT	1
	Irradiation dose	
	CT guidance: DLP (mGy.cm) (mean, SD, range)	1431 ± 1719 (196–6696)
	Fluoroscopy (FS):	
	DAP (mGy.cm)	52 ± 72 (3–245)
	Total dose (mGy)	189 ± 254 (8–753)
	Total FS time (s)	231 ± 242 (22–781)
	Length of stay (d) (median, IQR)	8 (1–31)
Benign	Type of needles	
	PR 15 or PR 20 (17 G)	6
	SR 25 (13 G)	1
	Power (W) (mean, SD, range)	33 ± 7 (30–50)
	Total time energy delivery (min) (mean, SD, range)	2.9 ± 2.5 (1.5–8.5)
	Biopsy	7
	Irradiation dose	
	CT guidance: DLP (mGy.cm) (mean, SD, range)	591 ± 498 (139–1525)
	Length of stay (d) (median, IQR)	1.7 (1–3)

*G* gauge, *W* watts, *SD* standard deviation, *min* minute, *RT* radiotherapy, *mGy.cm* milligray per cm, *s* second, *d* day, *IQR* interquartile, *DLP* dose length product, *DAP* dose area product

**Fig. 2:**
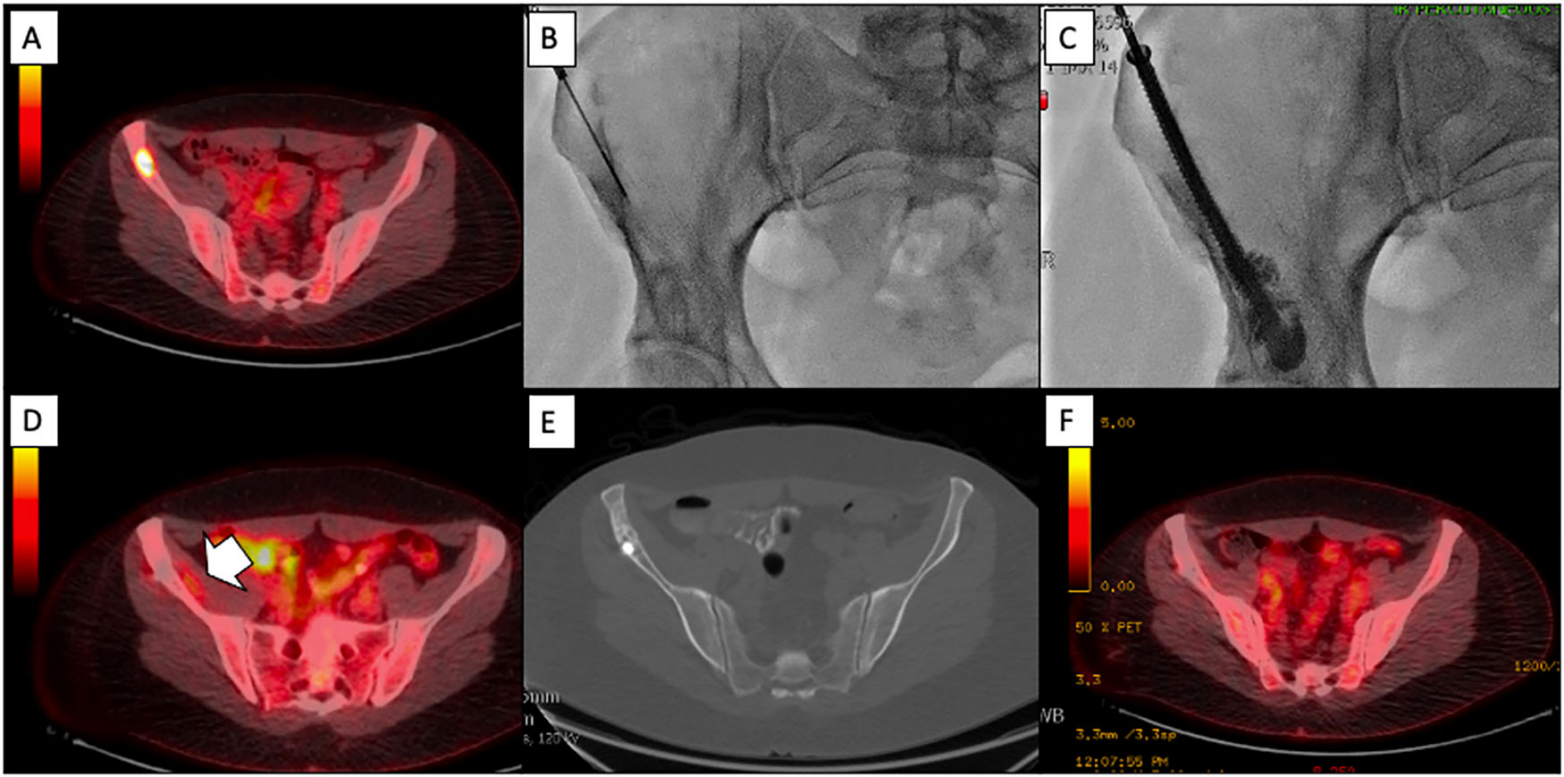
Pseudomyogenic hemangioendothelioma in a 40-year-old woman. **A** Pre-treatment PET scan showing the hypermetabolic lesion. **B**, **C** Fluoroscopic images during the MWA procedure (PR17, 60 W, 3 min) showing the positioning of the screw and cement. **D** PET scan at 3 months, indicating inflammation along the lesion margins. **E**, **F** PET scan at 30 months, with no signs of recurrence

Regarding the benign lesions, the average power delivered was 33 ± 7 watts [range: 30–50] with a mean total energy delivery time of 2.9 ± 2.5 min [range: 1.5–8.5]. A total of seven needles were used (one per lesion): six 17 gauge and one 13 gauge. Biopsies were conducted during the procedure for all patients. The average irradiation dose was 591 ± 498 mGy.cm [range: 139–1525]. The median hospital stay was 1.7 days [IQR: 1–3]. Figure 2 shows an example of procedures.

### Safety

Regarding metastatic lesions, 8.3% of patients (*n* = 3) experienced grade I adverse events (edema and temporary increased pain managed with morphine), and 2.8% (*n* = 1) experienced grade III adverse events (myonecrosis and complete radial nerve palsy). A pathological fracture occurred in the treated bone more than 1 month after the procedure (in a case where MWA was performed without osteoplasty), and was interpreted as disease progression rather than a procedural complication. No severe adverse events (grades 4–5) were reported. No adverse events were reported for patients with benign lesions.

### Efficacy

**Fig. 3:**
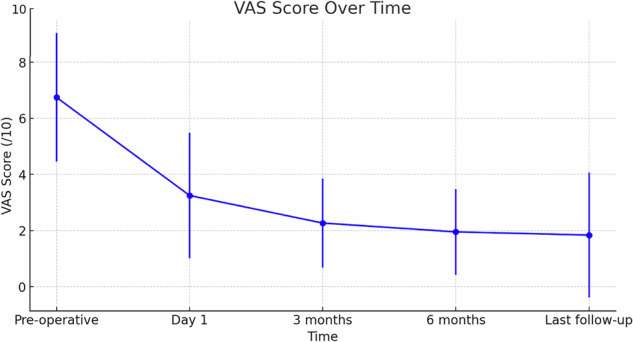
Graphical representation of mean and standard deviation of VAS scores before and after the procedure for metastatic patients

Regarding metastatic lesions, technical success was achieved on the first attempt in 100% of cases. The average follow-up was 16 ± 19 months [range: 1–87]. The median survival time was 9 months [IQR: 4–34], with 33.3% of patients alive at the last follow-up. The mean preoperative VAS score of 6.7 ± 2.3 (range: 0–10) significantly decreased at each time point after the operation (*p* < 0.001) to 1.8 ± 2.3 (range: 0–7) by the final follow-up (*p* < 0.001). The average VAS score remained stable from 3 months to the final follow-up post-operation, with no statistically significant fluctuations (Fig. 3). Postoperatively, on the SF-36 scale, 4 patients (11.1%) experienced no pain, 9 patients (25.0%) had very mild pain, 16 patients (44.4%) had mild pain, 4 patients (11.1%) had moderate pain, and 3 patients (8.3%) experienced severe pain. Twenty-nine (80.6%) patients reported improvement (very much or much improved) per the Patient Global Impression of Change. No patient had worsened pain. Complete response was achieved in 20 cases (55.6%), with a median time of recurrence of 13 months (IQR: 8–14).

In the subgroup analysis, lesions treated with both MWA and osteoplasty, as well as those treated with MWA, osteoplasty, and osteosynthesis, exhibited significantly higher pain levels at baseline compared to lesions treated with MWA alone (*p* = 0.026). However, there were no statistically significant differences in the VAS scores at 1 day (*p* = 0.267), 3 months (*p* = 0.095), 6 months (*p* = 0.101), or at the final follow-up (*p* = 0.151).

Regarding benign lesions, technical success was achieved on the first attempt in 100% of cases. The average follow-up was 8 ± 9 months [range: 2–29]. All patients were alive at the last follow-up. The mean preoperative VAS score of 5.7 ± 2.1 (range: 3–8) significantly decreased to 0 ± 0 (range: 0–0) by the final follow-up (*p* = 0.0011). On the SF-36 scale, all patients experienced no pain, and all were very much improved according to the Patient Global Impression of Change. Table 4 provides the VAS measurements, and Table 5 provides a detailed summary of the follow-up data.

**Table 4:** VAS measurements

	Pain intensity (VAS) Mean ± SD (range) median [IQR]	Global cohort	Subgroup without other procedure	Cementoplasty	Cementoplasty + percutaneous screw fixation	*p*-value*
Metastatic	Before operation	6.7 ± 2.3 (0–10) 7 [6–8]	6.4 ± 2.6 (0–9) 7 [6–8]	6.9 ± 2.1 (3–10) 7 [6–8]	8 ± 1.1 (7–10) 8 [7, 8]	0.026
	D1	3.5 ± 2.3 (0–8) 3 [2–4]	2.9 ± 2.1 (0–8) 3 [1–4]	4.2 ± 2.6 (0–8) 4 [2–5]	3.6 ± 0.8 (3–5) 3 [3, 4]	0.267
	M3	2.3 ± 1.6 (0–6) 2 [1–3]	2.1 ± 1.5 (0–4) 2 [0–4]	3.1 ± 1.7 (0–6) 3 [2–4]	3 ± 0.8 (2–4) 3 [2–4]	0.095
	M6	1.9 ± 1.6 (0–7) 2 [0–3]	1.5 ± 1.3 (0–4) 2 [0–2]	2.7 ± 1.5 (0–4) 3 [2–4]	3 ± 1.4 (1–5) 3 [2–4]	0.101
	Last follow-up	1.8 ± 2.3 (0–7) 1 [0–3]	1.1 ± 1.7 (0–6) 0 [0–2]	3.3 ± 2.2 (0–7) 2 [2–4]	2.7 ± 2.3 (0–7) 2 [2–4]	0.151
Benign	Before operation	5.7 ± 2.1 (3–8)	N/A	N/A	N/A	N/A
	M3	0 (0–0)	N/A	N/A	N/A	N/A
	Last follow-up	0 (0–0)	N/A	N/A	N/A	N/A

*VAS* visual analogic score, *IQR* interquartile range, *SD* standard deviation, *D* day, *M* month

* Differences between groups were tested using ANOVA. Shapiro–Wilk test assessing the normality of the score distributions. Following the ANOVA, a Tukey post hoc test was performed to identify specific group pairs with significant differences. Statistical significance was determined at a *p*-value of less than 0.05

**Table 5:** Follow-up

	Metastatic	Benign
Total duration (m) (mean, SD, range)	16 ± 19 (1–87)	8 ± 9 (2–29)
Adverse events	Grade I: 8.3% (3/36) Grade III: 2.8% (1/36)	0 0
Alive	33.3% (12/36)	100% (7/7)
Median survival time (m) (Median, IQR)	9 (4–34)	-
Median recurrence time (m) (Median, IQR)	13 (8–14)	-
Bodily pain score (SF-36)*		
None	4 (11.1%)	7 (100%)
Very mild	9 (25%)	0
Mild	16 (44.4%)	0
Moderate	4 (11.1%)	0
Severe	3 (8.3%)	0
Patient global impression change*		
Very much improved	8 (22.2%)	7 (100%)
Much improved	21 (58.3%)	0
Minimally improved	3 (8.3%)	0
No change	4 (11.1%)	0
Minimally worse	0	0
Much worse	0	0
Very much worse	0	0
Target response*		
CR	20 (55.6%)	7 (100%)
Not CR not PD	7 (19.4%)	0
PD	9 (25%)	0

*m* months, *CR* complete response, *PD* progressive disease

* at last follow-up

## Discussion

The safety profile of MWA is confirmed in this study by the low incidence of adverse events observed and is consistent with systematic reviews involving diverse percutaneous ablation techniques [12]. However, it is well known that MWA, as with any other ablation, affects bone remodeling by compromising the bone matrix and depleting the tissue of osteoblasts and osteoclasts. Therefore, to prevent displaced fractures, osteoplasty was offered, based on multidisciplinary review, in areas where compression stress occurred and fixation in case of shear stress, which limits the interpretation of the results.

MWA, similar to other techniques such as CA and RFA, leads to thermal necrosis of the tissue, effectively destroying periosteal nociceptors and eliminating cancerous cells [13]. It utilizes dielectric hysteresis to achieve direct volume heating of tissue, capable of propagating through various tissues, including those with high impedance such as bone. MWA is less susceptible to “heat sink” effects near vessels and allows for the creation of larger, customizable ablation zones in a shorter time frame, with minimal risk of the back-heating phenomenon in recently introduced antennas. It also has no contraindications for patients with metallic implants [10, 13]. Therefore, MWA offers several advantages over CA and RFA. MWA can generate higher temperatures more rapidly, leading to shorter ablation times and larger ablation zones, which is particularly beneficial for treating large or multiple bone lesions. Unlike RFA, the efficacy of MWA is not significantly affected by tissue impedance, which can vary in different bone environments [14]. However, the rapid delivery of high-power output can lead to less distinct ablation boundaries and a higher risk of nontarget overheating, notably when the cortex is compromised, potentially affecting surrounding tissues [14].

MWA provides a significant reduction in pain, as shown in this study, with a 74.3% decrease in VAS scores from preoperative levels to the last follow-up after MWA of bone metastases. The PGIC supports these results, with 80.6% of patients reporting substantial improvements post-procedure. Effective pain management not only alleviates physical discomfort but also reduces the psychological burden on patients [15–17]. These outcomes align with small studies published in the literature [18–20], such as Yang et al [21], where 88.9% of patients experienced post-operative pain relief in a cohort of 18 patients. While radiotherapy (RT) remains the gold standard for palliative treatment of painful bone metastases [22], 40 to 50% of the patients do not respond adequately [23], and most benefits are delayed, typically manifesting no sooner than 2 weeks post-treatment, with minimal improvements after 6 weeks [24]. Furthermore, RT is constrained by the radiation tolerance of surrounding normal tissues, limiting its applicability [25]. In this context, MWA may serve as a complementary or alternative therapy alongside RT. The results of MWA are similar to those of CA and RFA. A systematic review of CA, including 55 studies, showed a significant pain reduction after the procedure (mean difference between pre- and post-procedure VAS scores of 5.8 after 4 weeks), and an overall rate of minor and major adverse events of 12.7% [26]. In a large multicentric, prospective study (OsteoCool Tumor Ablation Post-Market Study), Levy et al [27] showed that RFA provides rapid (within 3 days) significant pain and quality of life improvements with sustained long-term relief through 12 months and a high degree of safety in lytic metastases treatment. No compelling indication of the superiority of one technique over another has been found [12].

However, the visibility of MWA on imaging, as with RFA, is very limited compared to CA, which can be directly monitored via real-time imaging modalities such as CT or MRI, allowing for precise control over the ablation zone [14]. There is a need to optimize treatment planning, delivery, and assessment as MWA’s visibility on imaging is limited. Integrating advanced imaging techniques, navigation, and robotics could help, but they need further evaluation. It would offer enhanced precision in targeting lesions, directly contributing to more accurate lesion ablations, while limiting impact on surrounding healthy tissues. This precision also significantly reduces procedure time and exposure to radiation for both patients and staff. Furthermore, using these tools can streamline the operational workflow, potentially increasing procedure efficiency and improving the overall safety of the process.

This study has limitations, including its monocentric, retrospective design and the heterogeneity of the metastatic primary tumors, which could introduce bias in efficacy outcomes. Additionally, the osteoplasty or fixation may also involve bias, as it reduces pain by itself. However, the use of cement was justified given the presence of fractures or the risk of fractures. The median hospital stay of 8 days observed in metastatic patients reflects not only the complexity of cases—including the frequent use of adjunctive procedures such as osteoplasty and fixation—but also organizational and social factors that affected discharge timing. This contrasts with shorter stays typically reported for isolated ablation procedures and underscores the importance of multidisciplinary coordination in complex bone interventions. Finally, this study focused exclusively on non-spinal bone lesions. The exclusion of spinal tumors was deliberate due to the specific technical considerations and higher risk of neurological complications associated with vertebral ablation. Although MWA has been investigated in the spine, its safety and efficacy in this context require dedicated evaluation, given the proximity to neural structures.

In conclusion, this study demonstrated the safety and efficacy of MWA as a treatment for local control and pain management in patients with bone metastases. MWA led to substantial pain reduction and improved quality of life. MWA represents a valuable alternative or complement for patients unsuitable for surgery or radiotherapy, and in selected cases with curative intent. Follow-up is reserved for asymptomatic or indolent tumors, while biopsy is indicated in cases of diagnostic uncertainty.

## Data Availability

Data is available from the authors upon reasonable request.

## Abbreviations

CA: Cryoablation
CT: Computed tomography
IQR: Interquartile range
MRI: Magnetic resonance imaging
MWA: Microwave ablation
PET: Positron emission tomography
PGIC: Patient Global Impression of Change
RFA: Radiofrequency ablation
RT: Radiotherapy
SF-36: Short Form-36
VAS: Visual Analogue Scale
